# Effects of high-protein diet combined with exercise to counteract frailty in pre-frail and frail community-dwelling older adults: study protocol for a three-arm randomized controlled trial

**DOI:** 10.1186/s13063-020-04572-z

**Published:** 2020-07-11

**Authors:** Sussi F. Buhl, Anne Marie Beck, Britt Christensen, Paolo Caserotti

**Affiliations:** 1 Centre for Active and Healthy Ageing, Department of Sports Science and Clinical Biomechanics, University of Southern Denmark, Campusvej 55, 5230 Odense, Denmark; 2 Department of Nutrition and Health, University College Copenhagen, Sigurdsgade 26, DK-2200 Copenhagen N, Denmark; 3 Dietetic and Nutritional Research Unit, Herlev-Gentofte University Hospital, DK-2730 Herlev, Denmark; 4 Arla Foods amba, Global Nutrition, Agro Food Park 19, 8200 Aarhus N, Denmark

**Keywords:** Frailty, Aging, High-protein diet, Protein supplementation, Resistance training, Strength training, Muscle power, Muscle strength, Muscle mass, Functional performance, Nutritional status

## Abstract

**Background:**

The proportion of older citizens is increasing worldwide. A well-known syndrome in old age is physical frailty which is associated with a greater risk of disabilities in activities of daily living, greater reliance on in-home services, hospitalization, institutionalization, and premature mortality. The purpose of this study is to determine the effects of an intervention with high-protein diet alone or in combination with power training in pre-frail and frail old adults.

**Methods:**

The study is a community-based assessor-blinded parallel randomized controlled trial (RCT), consisting of two phases. Phase 1 is a 1-month stabilization phase, where self-reliant community-dwelling adults + 80 years old will receive individual guidance regarding protein intake, to prevent the risk of negative protein balance prior to phase 2 and to only include participants who have reached the minimum recommended level of protein intake (1.0 g/kg/day) in the randomized controlled trial. Phase 2 is a 4-month RCT where 150 participants will be randomized into the following three arms: protein-only where participants will be provided with dairy products to increase their protein intake to 1.5 g/kg/day, protein + exercise where participants will be provided with the protein intervention in combination with power training two times a week, and recommendation group where participants will continue as in phase 1. Primary outcome is lower leg muscle power. Secondary outcomes include physical function and mobility, frailty status, muscle mechanical function, body composition, nutritional status, and health-related quality of life. The statistical analysis will include an intention-to-treat analysis of all randomized participant and per-protocol analysis of all compliant participants. The study hypothesis will be tested with mixed linear models to assess changes in the main outcomes over time and between study arms.

**Discussion:**

The finding of this study may add to the knowledge about the beneficial effects of high-protein diet from dairy products combined with power training to counteract frailty in community-dwelling older adults. This may ultimately have an impact on the ability to live well and independent for longer.

**Trial registration:**

ClinicalTrials.gov NCT03842579. Registered on 15 February 2019, version 1

## Background

The proportion of older citizens aged + 80 years old is increasing in the western population at faster rate than any other age group [1]. In Denmark, projections indicate that this group will represent 10% of the total population in 2060 from the current 4.4% [2].

Older age has been associated with greater prevalence of physical frailty a complex syndrome with multiple causes [3] characterized by reduced physiologic reserve and increased vulnerability to external and internal stressors [4]. The SHARE-FI75+ screening tool is specifically developed for + 75 years community-dwelling adults and operationalize non-frail, pre-frail, or frail condition by considering age, gender, and the presence of fatigue, low appetite, weakness, slowness, and low physical activity [5]. The prevalence of pre-frail and frail syndrome has been recently estimated as high as 43% between the age of 65 and 73 years using a slightly different screening tool earlier developed by Fried and colleagues [6], but data on the + 80 years old is still limited.

Physical frailty has been associated with greater risk of disabilities in basic and instrumental activities of daily living, chronic illnesses, loneliness, psychological distress, poorer self-reported quality of life, and premature mortality [7]. In addition, physical frail individuals have greater reliance on in-home services, risk for hospitalization, institutionalization, and overall greater health care cost [6, 8–11]. Hence, physical frailty has important implications for the individual older citizen as well as for the health care system.

One of the key determinants of this syndrome is the excessive loss of muscle mass which is strongly associated with the modifiable lifestyle factors, dietary protein intake and exercise [12, 13], both recognized as anabolic agents pivotal for primary, secondary, and tertiary prevention of physical frailty [14].

Specifically, diets high in protein are associated with a reduced risk of frailty [13]. Milk proteins are high-quality proteins with a high content of the amino acid leucine, known to effectively stimulate muscle protein synthesis [15, 16]. The current recommendation of total protein intake for older adults is minimum 1.0 g protein/kg/day based on European recommendations [12, 17], but it has been suggested that older adults, and especially frail older adults, can benefit from an even higher protein intake of 1.5 g protein/kg/day [18–20]. However, there is growing evidence that a large proportion of the older adults do not meet the recommendations for protein intake, e.g., due to anorexia of aging, medical conditions, and physical and mental limitations [12, 21, 22].

Exercise interventions focusing on strength and resistance training have been consistently shown to counteract the age-related decline of muscle function (e.g., muscle strength), physical function (e.g., walking speed) and disability among older adults [23, 24]. In addition, muscle power (the product of force times velocity) has been earlier considered a critical determinant to maintain function and independence in older age and exercise interventions targeting muscle power may play a key role in counteracting frail and pre-frail condition [25–27]. Several systematic reviews have investigated the beneficial effect of combining protein and exercise, but only one have examined the beneficial effect of protein alone [28] and none of the studies included in this review have used off-the-shelf dairy products or exercise interventions designed with muscle power components. Furthermore, it is unclear whether the combination of exercise and nutrition may be superior to the single interventions to improve physical performance in pre-frail and frail older adults as highlighted in a recent systematic review [29]. Possibly, the heterogeneity of the interventions (self-administered or supervised exercise programs, nutritional interventions range from supplements of selected micronutrients to protein supplementation from 10 to 30 g/day), duration, and settings (primary vs. secondary care settings) may have contributed to such unclear results [29]. Optimizing protein intake before initiating exercise may be essential for older adults who are not in protein balance to prevent the risk of an accelerated loss of muscle mass [30]. According to our knowledge, the benefit of optimizing protein intake before exercise interventions has not been examined before.

Therefore, the aims in this two phased parallel RCT is to investigate in pre-frail and frail older adults whether:

A. An increased protein intake from dairy products combined with power training increases (i) muscle power, and (ii) muscle mechanical function, maximal muscle strength, physical function, mobility, physical activity, muscle mass, health-related quality of life, and activities of daily living (iii) decrease pain, fear of falling, sedentary behavior, risk of malnutrition and (iv) modify physical frailty status and causes and contributors associated with such status.
B. Protein and power training in combination has a superior effect compared to an increased protein intake from dairy products alone and to a third intervention based on current recommendations on diet and physical activity for older adults.

## Methods

### Study design

**Fig. 1:**
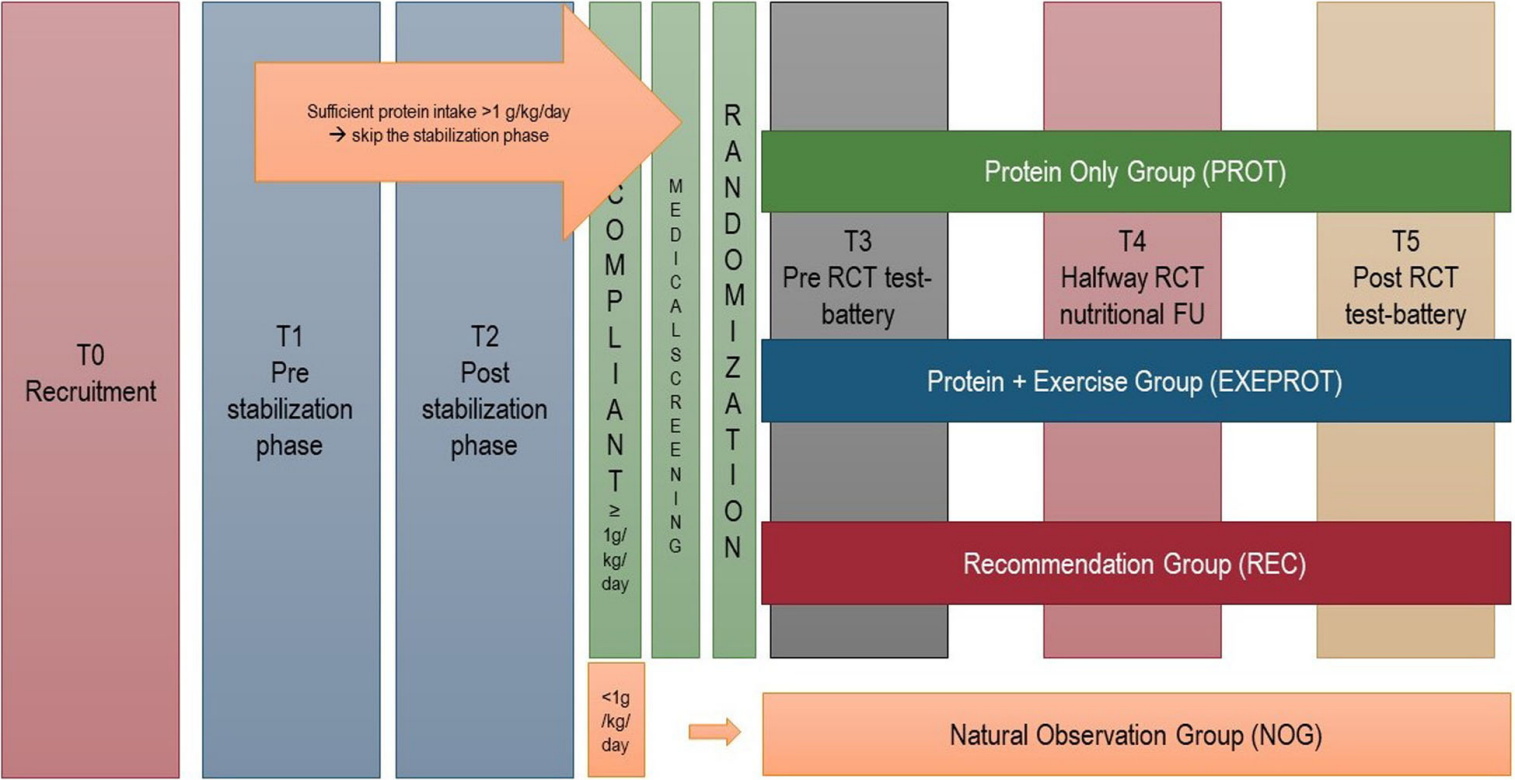
Flow-chart of the phases in the study (stabilization and intervention phases). RCT, randomized controlled trial; FU, follow-up; T, test session; g/kg/day, refers to g of protein/kg/day

A 2-phase community-based assessor-blinded parallel RCT will be undertaken, to determine the effects of interventions with off-the-shelf dairy products alone or in combination with progressive power training, see Fig. 1.

### Study procedure

#### Inclusion criteria for participants

Participants are required to be (1) community-dwelling older adults + 80 years old; (2) pre-frail or frail, evaluated by SHARE-FI75+ [5]; (3) intact cognitive function, evaluated by a score ≥ 4 in the short form of the Mini-Mental State Evaluation (MMSE) [31, 32]; (4) medically stable as evaluated by medical screening (see details below); (5) able to participate in group-based exercise without need for transportation or individual exercise sessions; (6) signed informed consent; (7) able to speak and read Danish; (8) not allergic/intolerant to milk-products/protein; and (9) not on a weight losing diet.

#### Exclusion criteria for participants

Participants are excluded if they are not medical stable, including (1) eGFR < 40 ml/min/1.73 m^2^; (2) have received cancer treatment during the preceding 6 months; (3) received high doses of prednisolone and/or morphine, evaluated by medical doctor; and (4) have other medical issues that may affect the study.

#### Procedure for recruitment

Participants will be recruited through 3 pathways:

1. Nationally regulated preventive home visits service managed by the municipality, as earlier reported in [33]. Community-dwelling adults ≥ 80 years who are self-reliant in activities of daily living are offered a home visit by health-care personnel at least once a year. During the home visit, the older citizens will be informed about the study. As part of the preventive initiatives, the municipality may also invite groups of older citizens to presentations on selected health topics. When related to physical function, nutrition, or exercise, the I’m still standing study will also be presented.
2. Existing cohort of community-dwelling older citizens (the Healthy Aging Network of Competence in Southern Denmark - Northern Schleswig-Holstein, HANC-study [33, 34]). All former participants will receive an invitation letter to the I’m still standing project.
3. Advertisements in newspapers, on webpages, and social medias and presentations about the project at arrangements targeting older adults. All advertisements will be performed in close collaboration between the University of Southern Denmark and the Municipality of Odense targeting community-dwelling adults + 80 years and their relatives or health care personnel in primary prevention.

Recruitment to the project via all pathways is performed in collaboration with the municipality of Odense. The health care personnel will perform the screening for inclusion criteria (apart from the medical screening) and obtain informed consent from eligible participants.

##### Phase 1: Stabilization phase—1-month nutritional recommendation

The aim of the stabilization phase (phase 1) is to ensure that participants enrolled in the intervention (phase 2) will have a minimum daily protein intake according to the European recommendation, ≥ 1.0 g/kg/day [12] to prevent the risk of negative protein balance.

Risk of protein malnutrition in this phase will be first operationalized by the Protein Screener (Pro55+) [35] administered during the first home visit. The Protein Screener classifies participants according to the probability of being protein malnourished, with a probability above 30% considered as cutoff point for insufficient intake of protein. Different scenarios may occur during this phase:

1. Participants classified to have high probability of protein malnutrition (> 30%) by the Pro55+ will be individually guided to improve their intake of protein and receive publicly available material regarding nutritional guidelines specifically developed for older adults. One month later, participants will be asked to fill out a 4-day food record (three weekdays and 1 day during the weekend) to evaluate if the minimum requirement for daily protein intake is met.
2. Participants classified to have a low probability of protein malnutrition (≤ 30%) by the Pro55+ will be immediately asked to fill out a 4-day food record (three weekdays and 1 day during the weekend). This step is to confirm that the average protein intake is ≥ 1.0 g/kg/day. If the 4-day food records indicate a protein intake below 1.0 g/kg/day, participants will be enrolled in the stabilization phase and an additional 4-day food records will be collected after 1 month.

Participants with a minimum intake of 1.0 g protein/kg/day will be invited to the medical screening. Participants not meeting the recommended protein intake after the stabilization phase will be followed as “natural observation group” but will not be considered for statistical purposes. They will receive two seminars on specific topics related to healthy aging and be asked to fill out self-reported questionnaires (at baseline and after 4 months) including demographic data, chronic diseases, activities of daily living, quality of life, appetite, nutritional status, sedentary behavior, function, pain and depression. Semi-structured interviews to identify potential barriers for not meeting the recommended level of daily protein intake will be conducted on a sub-group. The phases in the study are illustrated in Fig. 1.

#### Medical screening

The medical screening will be performed by a physician and will include blood pressure, auscultation of heart and lungs, blood sample, and an evaluation of medical history. Participants will be asked to bring their medications and direct count of name and dose will be performed. Medical stable is defined as no kidney diseases and eGFR ≥ 40 ml/min/1.73 m^2^, no cancer treatment within the last 6 months, no high doses of prednisolone and/or morphine, and no other medical issues that may affect the study.

##### Phase 2: Four months randomized controlled intervention

Eligible participants will be randomized into three groups: (i) protein-only group (PROT), (ii) protein + exercise group (EXEPROT), and (iii) recommendation group (REC).

1. PROT intervention

The PROT group will receive protein-rich off-the-shelf dairy products with the aim of targeting a daily protein intake of 1.5 g protein/kg/day. At baseline, individual supplementation plans are made based on information about participants habitual protein intake (assessed by the food records) and taste preferences. A cutoff of 15 g of supplementary protein is used—if participants have a higher requirement for protein, there will be a stepwise increase in protein intake over the first 4 weeks. Examples of products are skimmed milk, low-fat yoghurts with a high protein content (skyr), chocolate milk, cottage cheese, and ordinary low-fat cheese. Products will be delivered once a week at the home of the participant or at the training facilities. The type of products consumed may be changed throughout the study in order to avoid sensory-specific satiety and keep the compliance high. The PROT intervention is performed by specifically trained nutritional specialists.

2. EXEPROT intervention

The EXEPROT group will receive the same protein intervention as the PROT group combined with exercise program 1 h, twice a week. The exercise is designed as power training incorporating lower and upper body exercises with a training intensity of 65–80% of 1 repetition maximum (3 sets of 10–12 repetitions), which will be progressively adjusted throughout the entire intervention. Exercises will be performed explosively (i.e., as rapid as possible) during the concentric phase of the movement and controlled during the eccentric phase. At each session, participants register exercise activities, intensity, and rate of perceived exertion. The power training is performed by specially trained exercise specialists.

3. REC intervention

The REC group will be asked to follow the European recommendations for older adults on diet and physical activity over the course of the study and will be provided with publicly available material. In addition, two seminars on specific topics related to healthy aging will be provided to the group.

In all intervention groups, participants will continue with concomitant care and activities, e.g., if they have a rehabilitation plan including exercise. If participants experience changes in their medical condition, an additional consultation with the physician will be arranged to evaluate if participation in the study potentially will be harmful.

#### Compliance with the intervention

For the PROT and EXEPROT interventions, adherence to the protein protocol will be evaluated as average protein intake ≥ 1.35 g/kg/day at the 2 months and 4 months follow-up. Compliance and reasons for lack of compliance will also be estimated during each delivery of products with a set of questions (e.g., supplement consumption, changes to habitual food intake) and regular phone follow-ups. If participants are unable to reach the protein target, additional face-to-face or phone interview will be planned to support adherence. For the EXEPROT intervention, adherence to the exercise protocol will be considered as achieving minimum to 75% of valid exercise sessions, considered as minimum 70% of the exercises planned for each session. Reasons for non-adherence will be documented.

### Sample size determination

Muscle power is the primary outcome of this study. Due to lack of studies comparable to this study design (e.g., age and frailty status of the participants, type of exercise and level of protein supplementation), we have calculated sample size using a combination of studies and methods. Based on findings by Bechshøft et al. [36], the effect of 12 weeks of protein supplementation (two daily supplements of 20 g milk protein) in combination with resistance training in + 80-year-old healthy adults increased muscle power by 15% (SEM ± 5%) in comparison with − 7% (SEM ± 6%) in the control group (receiving protein supplementation only). In the study by Park et al. [37], 12 weeks of protein supplementation (0.8 g/kg/day, 1.2 g/kg/day, or 1.5 g/kg/day) to pre-frail or frail older adults above 70 years resulted in an increase in muscle mass (estimated by DXA) of approximately 4% in the group receiving 1.5 g/kg/day. Unpublished data from our own group shows that change in muscle mass (estimated by DXA) accounted for 1.95% of the change in muscle power (power-rig) in older adults following 12 weeks of explosive resistance training. Hence, the estimated effect of an increase in muscle mass of 4% on muscle power is 7.8%. Adding this to the results from Bechshøft et al. [36] gives us an estimated change on 0.8% in the PROT group. Assuming that the change in muscle mass are comparable in the three groups, we therefore expect a change in muscle power of 15%, 0.8%, and − 7% with a SD of 30 in the EXEPROT, PROT, and REC groups, respectively.

Setting a power of 0.8 a sample size with 37 participants in each arm should be enough to detect a significant difference in muscle power (significance level at 0.05). Adding 25% to account for dropouts a total of 150 participants is needed.

Strength calculation was made in PASS 14.

### Randomization and blinding

After the baseline assessment, each participant will be randomized to one of the three study groups (PROT, EXEPROT, or REC) using a computer-based random-block randomization scheme, clustering by couples when cohabitees wish to be enrolled together. The code is generated, concealed (sealed envelopes), and stored by personnel who is not part of run of the study. This personnel reveals the allocation to interventions based on ID numbers only to the research assistants who will inform participants. The trial has an open design with blind assessment of outcomes, which means that participants will be asked not to reveal group allocation when undergoing follow-up measurements, as researchers conducting follow-up measurements will be blinded to group allocation. To assess the extent to which blinding has been preserved, researchers will record the number of cases in which allocation was revealed.

### Outcome measures

A proposed schedule for enrolment, intervention, and assessment is shown in the Standard Protocol Items: Recommendations for Interventional Trials (SPIRIT), Table 1. In addition, recommended items to address for intervention trials are reflected in the SPIRIT Checklist [38] (Additional file 1) and in the WHO Trial Registration Data set (Additional file 2).

**Table 1:** Standard Protocol Items: Recommendations for Interventional Trials (SPIRIT) figure: proposed schedule for enrolment, intervention, and assessment

Time point		Study period					
	Enrolment	Stabilization phase	Allocation	Post-allocation			Close-out
	***t***_***1***_	***t***_***2***_	***t***_***3***_	***t***_***3***_	***t***_***4***_	***t***_***5***_	***t***_***5***_
**Enrolment**							
**Eligibility screen**	X						
**Informed consent**	X						
**Medical screening**			x				
**Nutrition check**	x	x			x		
**Allocation**			x				
Interventions*							
***[EXEPROT]***				↔	↔	↔	
***[PROT]***				↔	↔	↔	
***[REC]***					x	x	
**Assessments**							
***[Baseline variables, gender, age, education, marital status, depression, incontinence, chronic diseases, use of medication, risk of poor protein intake, eating symptom questionnaire, risk of dysphagia, appetite, dental status]***				X			
***[primary outcome variable lower leg muscle power]***				x		x	X
***[secondary outcome variables physical frailty status, jump muscle power, leg press, hand grip strength, SPPB, waist and hip circumference, body weight, lean mass, fat mass, BMD, health-related QoL, pain, fatigue, ADL, fear of falling, blood markers, physical activity, sedentary behavior, nap and sleep, dietary intake, walking speed, rising from laying position, MNA, EVS, risk of poor protein intake, DDST]***				x		x	x

*DSST* Digit Symbol Substitution Test, *SPPB* Short Physical Performance Battery, *BMD* bone mineral density, *QoL* quality of life, *ADL* activities of daily living, *MNA* Mini Nutritional Assessment, *EVS* Eating Validation Scheme

*See explanation in the text

#### Test sessions

Five test sessions will be performed (T1–T5), see Fig. 1 and Table 1.

T1, T2 and T4: The pre- and post-test of the stabilization period and the test halfway through the intervention will consist of a nutrition check including assessment of protein intake by Pro 55+, 4-day food records, and 24-h recall interviews.

T3 and T5: The pre- and post-intervention test will include assessment of baseline variables and primary and secondary outcomes (specified in Table 2).

**Table 2:** An overview of variables and outcomes, outcome measures, instruments and time point for the assessment

Outcome	Outcome measures	Instrument	Time point
**Descriptive variables**			
Personal information	Age, gender, former job, marital status, educational background and chronic diseases	Self-report	T3
	Depression	The Major Depression Inventory (MDI) [39]	T3
	Incontinence	International Consultation on Incontinence Questionnaire (ICIQ) [40]	T3
	Eating ability	Self-reported dental state	T3
		The Eating Symptom Questionnaire [41]	
		The EAT-10 Questionnaire [42]	
		Simplified Nutritional Appetite Questionnaire [43]	
**Primary outcome**			
Muscle power	Lower leg muscle power	The Nottingham Leg Rig [44–46]	T3 and T5
**Secondary outcomes**			
Muscle mechanical function	Countermovement jump	The countermovement jump is performed on a force platform (Kistler 9281 B, 40 × 60 × 5 cm) following the procedure described in [47]. Four maximal jumps will be performed with 1-min interval and the highest jump recorded	T3 and T5
Maximal muscle strength	Leg press	Assessed on the dominant leg in a custom-built unilateral leg press device with a fixed footplate instrumented with piezoelectric force transducers (Kistler 9367/8 B). The force signals will be digitally sampled at 1 kHz while on-line visual feedback is provided to the subject. The contractile rate of force development and impulse will be determined in the trial with the highest resultant peak force [44, 48]	T3 and T5
	Handgrip strength	Handgrip strength is measured using a handheld dynamometer (Original Smedley’s Daynameter, Scandidact, 100 kg, Cat. No. 281128). Participants are instructed to sit with the elbow at a 90° angle, the wrist in neutral position. The inner lever of the dynamometer is adjusted to the hand of the participants (the second phalanxes against the lever) (Andersen-Ranberg et al. 2009). A minimum of three contractions in each hand will be performed and testing continues until participant produce less force than the prior test.	T3 and T5
Physical Frailty Status	The SHARE-FI75+ Fried frailty phenotype	The SHARE-FI75+ is a physical frailty assessment tool that is developed specifically for community-dwelling adults aged ≥ 75 years [5]	T3 and T5
		Fried frailty phenotype consists of five variables where three are based on questions: (i) unintentional weight loss, (ii) self-reported exhaustion, and (iii) low energy expenditure and the remaining two are based on objective assessment: (iv) slow gait speed and (v) weak hand grip strength [9]. The variable ‘low energy expenditure’ is modified to follow the current recommendations on physical activity in older adults from the World Health Organization [49].	
Risk of malnutrition	Protein intake	The Protein Screener Pro55+ is used to assess the risk of poor protein intake [35]	T1, T2, T3 and T5
		Four days food records (filled out on three weekdays and one day during the weekend) are calculated (Winfood 4.1) to estimate the average protein intake (g) per kg body weight per day and protein content per meal. In addition, energy intake and distribution of macronutrients are calculated.	
	Weight loss	Self-reported unintentional weight changes during the last month	T1, T2, T3, T4, and T5
	Nutritional status	The Eating Validation Scheme (EVS) is composed of five questions about dietary intake and weight loss and three questions about risk factors (dysphagia, eating assistance, and acute illness) [50]	T3 and T5
		The Mini Nutritional Assessment (MNA) is composed of 18 questions and measurements concerning appetite, eating ability, weight, need for help, illness, and medication [51]	
Anthropometry	Weight	Measured in light clothes, without shoes and subtracting 0.5 kg for the weight of clothes using a calibrated TANITA scale (model DC430SMA)	T3 and T5
	Waist- and Hip- circumference	Following the protocol by the World Health Organization [52]	
	Height	Measured without shoes	T3
Body composition	Estimation of fat mass, fat-free mass and bone mass	Dual-energy X-ray absorptiometry (DXA) (Lunar Prodigy) scans will be used to assess whole body composition with special emphasis on lean mass and fat tissue as well as bone mineral density.	T3 and T5
		Foot-to-foot bioelectrical impedance analysis (BIA), using the TANITA Total Body Composition Analyzer (model DC430SMA).	
Physical function and mobility	Gait speed, Chair stand, Balance	The Short Physical Performance Battery (SPPB) [53]	T3 and T5
		Rising from laying position on the floor and stand and reach test [54]	
		Self-selected and maximal gait speed (10 m) [55–57]	
		Distance walked during 2 min [58, 59]	
Physical activity level	Objective measures of intensity, duration, and patterns of physical activity and sedentary behavior	Hip-worn accelerometers, ActiGraph (removed during sleep)	T3 and T5
		Thigh-worn accelerometers, Axivity (24 h/day)	
		Data will be continuously collected in a raw format at 30/50 Hz, respectively, over a period of 7 days.	
	Self-reported sedentary behavior	The Sedentary Behavior Questionnaire (SBQ) [60]	T3 and T5
Activities of daily living	Self-report	Questionnaire that combines items from the Most Efficient Lists and the Short-Form of Late-Life Function and Disability Instrument [61–63]	T3 and T5
Pain, fatigue, and fatigability	Self-report	The Brief Pain Inventory - Short Form (BPI-sf) [64]	T3 and T5
		The Mobility fatigue scale “Mob-T” [65]	
		The Pittsburgh Fatigability Scale (PFS) for older adults (only the domain about physical fatigue will be assessed) [66]	
Fear of falling	Self-report	The Falls Efficacy Scale – International (FES-I) [67]	T3 and T5
		Self-reported falls within the last year	
Cognitive function	Pencil and paper test	The Digit Symbol Substitution Test (DSST) [68, 69]	T3 and T5
Health-related quality of life	Self-report	The EQ-5D-3L questionnaire [70]	T3 and T5
Biomarkers	Blood sample	Biomarkers related to nutritional status, physical activity, sedentary behavior, as well as physical and cognitive function—such as lipids, hormones, proteins, cytokines, vitamins, and minerals, e.g., cholesterol, HbA1c, adiponectin, suPAR, Crp, IL-6, B_12_ and vitamin D, vitamin B_12_ [71–74]	T3 and T5

*T1* time of enrolment, *T2* post the stabilization phase, *T3* baseline pre-intervention, *T4* half-way follow-up, *T5* close-out post-intervention

#### Data collection

Collection of data will be performed by trained research assistants from the University of Southern Denmark. Training of the research assistants includes theoretical teaching in the applied methods and tests, supervision by other research assistant, and frequent meetings with colleagues to share experiences and answers any questions that may arise. The descriptive variables (personal information, information about chronic diseases, depression, incontinence and eating ability) will be collected at baseline (T3). The primary and secondary outcomes data will be collected at baseline pre-intervention (T3) and post-intervention (T5). Participants will be invited to test irrespectively of their compliance to the intervention protocol. Participants are offered personal feedback on selected outcomes after ending the interventions to promote retention in intervention and post-intervention tests (T5).

### Primary outcome

Lower leg muscle power is the primary outcome. This will be assessed unilaterally using the Nottingham Leg Rig [44–46] on the dominant leg. A minimum of six trials will be conducted with approximately 45 s of rest between trials. Testing continues until participants produce less power than the prior test.

### Secondary outcomes

The secondary outcomes include assessment of muscle mechanical function, maximal muscle strength, physical frailty status, protein intake, risk of malnutrition, anthropometry, body composition, physical function, mobility, physical activity, sedentary behavior, pain, fatigue, fear of falling, cognitive function, health-related quality of life, and biomarkers.

For an overview of descriptive variables, outcomes, instruments, and assessment time points, see Table 2.

### Statistical analysis

Plans for data entry, coding, security, and storage including any related processes to promote data quality have been approved by the Danish Regional Data Protection Agency. The full analysis set will follow an intention-to-treat principle and will include all randomized participants. The study hypothesis will be tested with mixed linear models to assess changes in the main outcomes over time and between study arms controlling for confounders (e.g., age, sex, lifestyle factors). Per-protocol analysis of participants that are compliant to the protocol will be performed. Lastly, if sample size allows it, sub-group analysis by sex and by habitual lifestyle (protein intake and physical activity) will be performed based on baseline assessment. Wilcoxon signed rank sum test, Mann-Whitney *U* test, and odds ratio or chi-square test of relationships were used when appropriate. The statistical software programme STATA 16 (StataCorp. 2019. *Stata Statistical Software: Release 16*. College Station, TX: StataCorp LLC) will be used for the statistical analysis.

## Discussion

This study provides a protocol for a community-based assessor-blinded RCT to determine the effects of interventions with high-protein diet alone or in combination with power training on muscle mechanical function (muscle power and strength), frailty status, functional performance, muscle mass, and quality of life. This may ultimately have an impact on the ability of older pre-frail and frail adults to maintain self-reliance for longer.

Currently, only few of the studies that have investigated the effect of combining protein and exercise have used individually assigned supplementation plans that take the participants’ habitual protein intake and preferences into account. Specifically, in relation to pre-frail or frail older adults, most former multidomain studies have focused on increasing intake of protein by means of supplements (protein powder or oral nutritional supplements) without individual adjustments in doses. In addition, several studies have been designed with resistance training protocol, but only few studies have used power training combining heavy loading with maximum intentional acceleration of the training load—and, to our knowledge, none of them in combination with a nutritional intervention. Further, none of the studies seemed to focus on optimizing the protein balance before starting the exercise all which contrasts with the present study.

Even though the current recommendation of total protein intake for older adults is ≥ 1.0 g protein/kg/day based on European recommendations [17], it has been suggested that older adults, and especially frail older people, can benefit from an even higher protein intake of 1.5 g protein/kg/day [18, 19]. To evaluate the effects of a higher intake in comparison with the current recommendation, we have included the REC group as one of the intervention arms (illustrated in Fig. 1).

### Study limitations

The study faces several important challenges. One of the major challenges is recruitment, compliance with supplementation, and adherence to the exercise intervention because of the age (80+) and the pre-frail and frail syndrome of the participants. In addition, the high number of tests in the study may increase the risk of dropouts. In order to limit the impact of these challenges, recruitment is performed in collaboration with health care personnel from the department for the preventive home visits in the municipality of Odense. They are experienced in addressing this population and can contact community-dwelling + 80-year-old citizens that live within a geographically limited area. This gives the opportunity to offer exercise intervention in a local facility and to deliver the products directly to the homes of the participants and thereby limiting the impact of need for transportation. To limit the risk of dropouts during the study, participants are carefully informed about the different tests before and during the study. Participants are informed that they can always decline some of the tests and still be part of the study.

The 4-day food records are important components of this study both in phase 1 and phase 2. The method is demanding for the participants, and the self-reported intake may be affected by social desirability and memory impairments. This may translate into under- or overreporting and result in misclassification of protein status (phase 1) and miscalculation of additional need for protein (phase 2). Nevertheless, the method has been used and validated in other studies among older adults [75, 76]. In addition, the food records are qualified together with the participant during a face-to-face interview and supplemented with the probability scores from the Pro55+ [35].

The power calculation assumes that EXEPRROT is superior to PROT which is superior to REC. Since no former studies have used a study design comparable to the one used in the current study, we needed to base our power calculation on a combination of two studies. Furthermore, the power calculation is based on the number of participants needed for phase 2 of the study. However, it is unknown how many participants we have to include in phase 1 to find the 150 participants that have the required protein intake of 1 g/kg/day. To decrease the risk of having to recruit a very large sample, each participant will receive individual nutritional guidance in phase 1, together with written information about protein-rich diets specifically developed for our target group.

### Study strengths

A major strength of the study is the design with recruitment of the participants in collaboration with the preventive home visit service of the municipality of Odense. Different recruitment pathways are used for recruitment in agreement with Danish National law. This dictates that the preventive home visit service should identify older adults at risk of functional loss and disability using original pathway (e.g., directly contact by phone and invitation letter to older adults who are 80+) as well as additional ad hoc pathways to reach older adults who may be eligible (pre-frail/frail) but are more difficult to enroll. This design allows us to study the effects of a targeted action plan developed in collaboration with primary preventive sector to community-dwelling citizens in a community setting.

Furthermore, we design a “decentralized” intervention model where the exercise facilities are close to the older citizen and require minimal transport. Selection bias cannot completely be ruled out, but the specific design of the study will contribute to minimize it. According to our knowledge, such a study set-up with a strong applied element has never been used before. If the used interventions appear to be effective, they may rapidly be implemented in the primary preventive sector.

Another strength is the exercise intervention applied in the study. The intervention is supervised by skilled instructors, but it is also carried out in the municipality and hence will not demand other than the available equipment which may ease the implementation (if proven successful). The REC group will receive available information about the recommendations on nutrition and physical activity in older age in addition to two talks about these topics. This resemble the current practice in many municipalities and makes the control group very realistic, which helps to illustrate the additional effect of a more targeted intervention.

In contrast to former studies, we have chosen to provide the participants in the EXEPROT and PROT with ordinary off-the-shell dairy-based protein-rich products. This type of intervention has several advantages: off-the-shelf products are cheap, known by the participants, there is a large range of products, and they are easy to buy—this may increase compliance as participants recognize the products, are less likely to experience sensory-specific satiety, and (if proven successful) make it easy to transfer into recommendations and apply. Further, if the PROT arm show an effect of only adding protein, this may be of relevance to several older adults who, due to severe physical limitations or other, are unable to participate in exercise interventions.

The intervention period is 16 weeks which in general is slightly longer than several intervention studies. The longer intervention period gives the opportunity to have a familiarization period to the interventions (stepwise increase in protein intake, focus on technique in the exercise sessions), which may increase compliance to the protein intervention and reduce the risk of injuries from the exercise intervention. In addition, a recent meta-analysis have illustrated that the combination of protein supplementation and exercise is superior to exercise alone, but longer interventions (> 12 weeks) are required in the population of older adults [77].

The stabilizations phase provides us with a lot of important knowledge about the participants “starting point” and makes it possible to investigate who benefits most from the intervention—the ones with a habitual high intake or the ones who largely increase their intake of protein? Also, we can investigate who is able to increase their intake and what are the barriers for not increasing.

In general, our initiatives to monitor protein intake and compliance with the power training might help us to understand who benefits, how much is needed, and how the results are affected by participants habitual habits in relation to dietary protein intake and physical activity.

In summary, this study will add important knowledge to understand the influence of protein supplementation alone with off-the-shelf dairy products or combined with structured power training on physical frailty. Developing action plans which counteract physical frailty is extremely important to increase the proportion of adults + 80 years who remain self-reliant. Maintaining the ability to perform activities of daily living is essential both for older citizens as well as for health care providers.

### Trial status

Recruitment of intervention and control group participants was still ongoing at the time of manuscript submission. The protocol version number is NCT03842579 (15 February 2019), the date recruitment began was 13 February 2019, and the approximate date when recruitment will be completed is 01 June 2021.

## Supplementary information

**Additional file 1.** The SPIRIT 2013 Checklist.

**Additional file 2:.** the WHO Trial Registration Data Set.

## Data Availability

Not applicable, no datasets are included in this study protocol.

## Abbreviations

RCT: Randomized controlled trial
Pro55+: Protein Screener
PROT: Protein-only group
PROTEXE: Protein and exercise group
REC: Recommendation group
NOG: Natural observation group
MMSE: Mini Mental State Examination
DXA: Dual-energy X-ray absorptiometry
QoL: Quality of life
SPPB: Short Physical Performance Battery
EVS: Eating Validation Scheme
MNA: Mini Nutritional Assessment
