# Supplementary material for: Effects of high-protein diet combined with exercise to counteract frailty in pre-frail and frail community-dwelling older adults: study protocol for a three-arm randomized controlled trial
Source: Trials. 2020 Jul 11;21:637. doi: 10.1186/s13063-020-04572-z (PMC7353704; doi:10.1186/s13063-020-04572-z)
Supplement: Supplementary file 1 — Additional file 1. The SPIRIT 2013 Checklist. [file 13063_2020_4572_MOESM1_ESM.docx]

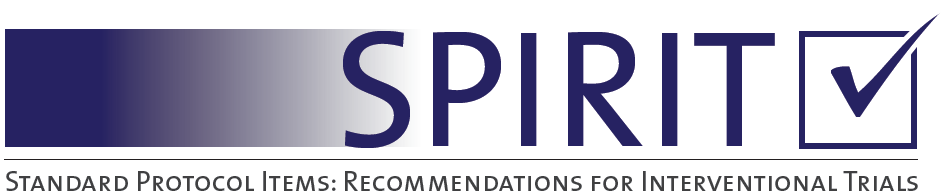


# Additional file 1 – The SPIRIT 2013 Checklist

SPIRIT 2013 Checklist: Recommended items to address in a clinical trial protocol and related documents*

| Section/item | Item No | Description |
| --- | --- | --- |
| **Administrative information** | | |
| Title | 1 | Descriptive title identifying the study design, population, interventions, and, if applicable, trial acronym – see page 1 |
| Trial registration | 2a | Trial identifier and registry name. If not yet registered, name of intended registry – the trial is registered in Clinical Trials Gov, see page 3 |
|  | 2b | All items from the World Health Organization Trial Registration Data – attached to the study protocol in additional file 2 |
| Protocol version | 3 | Date and version identifier - stated in the Abstract, see page 3 |
| Funding | 4 | Sources and types of financial, material, and other support – see the section ‘Funding’ at page 22 |
| Roles and responsibilities | 5a | Names, affiliations, and roles of protocol contributors – stated on the title page, page 1, and under the section ‘Authors contributions’, page 24 |
|  | 5b | Name and contact information for the trial sponsor – the trial sponsor is indicated on the title page, page 1. |
|  | 5c | Role of study sponsor and funders, if any, in study design; collection, management, analysis, and interpretation of data; writing of the report; and the decision to submit the report for publication, including whether they will have ultimate authority over any of these activities – see the section ‘Funding’, page 22. |
|  | 5d | Composition, roles, and responsibilities of the coordinating centre, steering committee, endpoint adjudication committee, data management team, and other individuals or groups overseeing the trial, if applicable (see Item 21a for data monitoring committee) – See the section ‘Roles and Responsibilities’, page 23 |
| Introduction |  |  |
| Background and rationale | 6a | Description of research question and justification for undertaking the trial, including summary of relevant studies (published and unpublished) examining benefits and harms for each intervention – See the Background section, page 4-6 |
|  | 6b | Explanation for choice of comparators – see page 4-6 |
| Objectives | 7 | Specific objectives or hypotheses – see the aims in the end of the background section, page 5-6 |
| Trial design | 8 | Description of trial design including type of trial (eg, parallel group, crossover, factorial, single group), allocation ratio, and framework (eg, superiority, equivalence, noninferiority, exploratory) – see section ‘Study design’, page 6, and ‘Randomization and blinding’, page 11. |
| Methods: Participants, interventions, and outcomes | | |
| Study setting | 9 | Description of study settings (eg, community clinic, academic hospital) – see section ‘Study design’, page 6 |
| Eligibility criteria | 10 | Inclusion and exclusion criteria for participants – See section ‘Study Procedure’, ‘Inclusion criteria for participants’, page 6 |
| Interventions | 11a | Interventions for each group with sufficient detail to allow replication, including how and when they will be administered – see the sections ‘Phase 1: stabilization phase – one-month nutritional recommendation’, ‘Phase 2: four months randomized controlled intervention’ pages 7-10 |
|  | 11b | Criteria for discontinuing or modifying allocated interventions for a given trial participant (eg, drug dose change in response to harms, participant request, or improving/worsening disease) – see the section ‘Phase 2: four months randomized controlled intervention’, page 10 |
|  | 11c | Strategies to improve adherence to intervention protocols, and any procedures for monitoring adherence (eg, drug tablet return, laboratory tests) – see section ‘Phase 2: four months randomized controlled intervention’, page 9-10 |
|  | 11d | Relevant concomitant care and interventions that are permitted or prohibited during the trial – see section ‘Phase 2: four months randomized controlled intervention’, page 10 |
| Outcomes | 12 | Primary, secondary, and other outcomes, including the specific measurement variable (eg, systolic blood pressure), analysis metric (eg, change from baseline, final value, time to event), method of aggregation (eg, median, proportion), and time point for each outcome. Explanation of the clinical relevance of chosen efficacy and harm outcomes is strongly recommended – see section ‘Outcome measures’ & Table 2, page 11-17 |
| Participant timeline | 13 | Time schedule of enrolment, interventions (including any run-ins and washouts), assessments, and visits for participants. A schematic diagram is highly recommended – see Figure 1 and Table 1 |
| Sample size | 14 | Estimated number of participants needed to achieve study objectives and how it was determined, including clinical and statistical assumptions supporting any sample size calculations – see section ‘Sample size determination’ page 10-11 |
| Recruitment | 15 | Strategies for achieving adequate participant enrolment to reach target sample size – see section ‘Study Procedure’ – ‘Procedure for recruitment’ page 6-7 |
| **Methods: Assignment of interventions (for controlled trials)** | | |
| Allocation: |  |  |
| Sequence generation | 16a | Method of generating the allocation sequence (eg, computer-generated random numbers), and list of any factors for stratification. To reduce predictability of a random sequence, details of any planned restriction (eg, blocking) should be provided in a separate document that is unavailable to those who enrol participants or assign interventions – see section ‘Randomization and blinding’ page 11 |
| Allocation concealment mechanism | 16b | Mechanism of implementing the allocation sequence (eg, central telephone; sequentially numbered, opaque, sealed envelopes), describing any steps to conceal the sequence until interventions are assigned - see section ‘Randomization and blinding’ page 11 |
| Implementation | 16c | Who will generate the allocation sequence, who will enrol participants, and who will assign participants to interventions – see section ‘Randomization and blinding’, page 11 |
| Blinding (masking) | 17a | Who will be blinded after assignment to interventions (eg, trial participants, care providers, outcome assessors, data analysts), and how – see section ‘Randomization and blinding’ page 11 |
|  | 17b | If blinded, circumstances under which unblinding is permissible, and procedure for revealing a participant’s allocated intervention during the trial - see section ‘Randomization and blinding’ page 11 |
| **Methods: Data collection, management, and analysis** | | |
| Data collection methods | 18a | Plans for assessment and collection of outcome, baseline, and other trial data, including any related processes to promote data quality (eg, duplicate measurements, training of assessors) and a description of study instruments (eg, questionnaires, laboratory tests) along with their reliability and validity, if known. Reference to where data collection forms can be found, if not in the protocol – see section ‘Outcome measures’ pages 11-17, Figure 1, Table 1 and Table 2 |
|  | 18b | Plans to promote participant retention and complete follow-up, including list of any outcome data to be collected for participants who discontinue or deviate from intervention protocols – see section ‘Data collection’, page 13-14 |
| Data management | 19 | Plans for data entry, coding, security, and storage, including any related processes to promote data quality (eg, double data entry; range checks for data values). Reference to where details of data management procedures can be found, if not in the protocol – see section ‘Statistical analysis’, page 17 |
| Statistical methods | 20a | Statistical methods for analysing primary and secondary outcomes. Reference to where other details of the statistical analysis plan can be found, if not in the protocol – see section ‘Statistical analysis’, page 17 |
|  | 20b | Methods for any additional analyses (eg, subgroup and adjusted analyses) – see section ‘Statistical analysis’, page 17 |
|  | 20c | Definition of analysis population relating to protocol non-adherence (eg, as randomised analysis), and any statistical methods to handle missing data (eg, multiple imputation) – see section ‘Statistical analysis’, page 17 |
| **Methods: Monitoring** | | |
| Data monitoring | 21a | Composition of data monitoring committee (DMC); summary of its role and reporting structure; statement of whether it is independent from the sponsor and competing interests; and reference to where further details about its charter can be found, if not in the protocol. Alternatively, an explanation of why a DMC is not needed – in this study a DMC is not needed due to the relatively short duration of the study. In addition, the interventions are known to have minimal risks for the participants. |
|  | 21b | Description of any interim analyses and stopping guidelines, including who will have access to these interim results and make the final decision to terminate the trial – no interim analyses will be performed in this study due to the minimal risks related to the interventions. |
| Harms | 22 | Plans for collecting, assessing, reporting, and managing solicited and spontaneously reported adverse events and other unintended effects of trial interventions or trial conduct – see section ‘Ethical approval and consent to participate’, page 22 |
| Auditing | 23 | Frequency and procedures for auditing trial conduct, if any, and whether the process will be independent from investigators and the sponsor – No audits has been planned in this single-site study |
| Ethics and dissemination | | |
| Research ethics approval | 24 | Plans for seeking research ethics committee/institutional review board (REC/IRB) approval – The study has been approved by the Regional Committees on Health Research Ethics for Southern Denmark, see section ‘Ethical approval and consent to participate’, page 22 |
| Protocol amendments | 25 | Plans for communicating important protocol modifications (eg, changes to eligibility criteria, outcomes, analyses) to relevant parties (eg, investigators, REC/IRBs, trial participants, trial registries, journals, regulators) - see section ‘Ethical approval and consent to participate’, page 22 |
| Consent or assent | 26a | Who will obtain informed consent or assent from potential trial participants or authorised surrogates, and how (see Item 32) – see section ‘Procedure for recruitment’, page 6-7 |
|  | 26b | Additional consent provisions for collection and use of participant data and biological specimens in ancillary studies, if applicable – see section ‘Procedure for recruitment’, page 6-7 |
| Confidentiality | 27 | How personal information about potential and enrolled participants will be collected, shared, and maintained in order to protect confidentiality before, during, and after the trial – see section ‘Ethical approval and consent to participate’, page 22 |
| Declaration of interests | 28 | Financial and other competing interests for principal investigators for the overall trial and each study site – see sections ‘Competing interests’ and ‘ Funding’ page 22-23 |
| Access to data | 29 | Statement of who will have access to the final trial dataset, and disclosure of contractual agreements that limit such access for investigators – No datasets are included in this study protocol |
| Ancillary and post-trial care | 30 | Provisions, if any, for ancillary and post-trial care, and for compensation to those who suffer harm from trial participation – see section ‘Ethical approval and consent to participate’, page 22 |
| Dissemination policy | 31a | Plans for investigators and sponsor to communicate trial results to participants, healthcare professionals, the public, and other relevant groups (eg, via publication, reporting in results databases, or other data sharing arrangements), including any publication restrictions – see section ‘Ethical approval and consent to participate’, page 22 & ‘Funding’, page 22-23 |
|  | 31b | Authorship eligibility guidelines and any intended use of professional writers – see section ‘Ethical approval and consent to participate’, page 22 |
|  | 31c | Plans, if any, for granting public access to the full protocol, participant-level dataset, and statistical code – Data collected during this study will be managed, stored and shared according to our approval from the to the Research & Innovation Organization at the University of Southern Denmark & following the Danish Legislation for Data Protection. Hence, personal data will be deleted, anonymized or archived no later than the 31^st^ of December 2029. |
| Appendices |  |  |
| Informed consent materials | 32 | Model consent form and other related documentation given to participants and authorised surrogates – As described in the section ‘Ethical approval and consent to participate’ a written consent from the Regional Committees on Health Research Ethics for Southern Denmark and from the Research & Innovation Organization at the University of Denmark will be applied. The forms are not attached here as they are in Danish but can be send if requested. |
| Biological specimens | 33 | Plans for collection, laboratory evaluation, and storage of biological specimens for genetic or molecular analysis in the current trial and for future use in ancillary studies, if applicable – No genetic analysis will be performed in this study. |

*It is strongly recommended that this checklist be read in conjunction with the SPIRIT 2013 Explanation & Elaboration for important clarification on the items. Amendments to the protocol should be tracked and dated. The SPIRIT checklist is copyrighted by the SPIRIT Group under the Creative Commons “[Attribution-NonCommercial-NoDerivs 3.0 Unported](http://www.creativecommons.org/licenses/by-nc-nd/3.0/)” license.
