# Supplementary material for: Effects of high-protein diet combined with exercise to counteract frailty in pre-frail and frail community-dwelling older adults: study protocol for a three-arm randomized controlled trial
Source: Trials. 2020 Jul 11;21:637. doi: 10.1186/s13063-020-04572-z (PMC7353704; doi:10.1186/s13063-020-04572-z)
Supplement: Supplementary file 2 — Additional file 2:. the WHO Trial Registration Data Set. [file 13063_2020_4572_MOESM2_ESM.docx]

# Additional file 2 - the WHO Trial Registration Data Set

| **Data category** | **Information** |
| --- | --- |
| Primary registry and trial identifying number | ClinicalTrials.Gov  NCT03842579 |
| Date of registration in primary registry | 15.02.19 |
| Secondary identifying numbers | S-20180048  10.122 |
| Source(s) of monetary or material support | Danish Dairy Research Foundation |
| Primary sponsor | Centre for Active and Healthy Ageing, Institute of Sports Science and Clinical Biomechanics, University of Southern Denmark |
| Secondary sponsor(s) | Department of Nutrition and Health, University College Copenhagen  Arla Foods Amba, Global Nutrition. |
| Contact for public queries | SFB, AMB, BC & PC |
| Contact for scientific queries | SFB & PC |
| Public title | Prevention of physical frailty in community-dwelling adults +80 years |
| Scientific title | Effects of high-protein diet combined with exercise to counteract frailty in pre-frail and frail community-dwelling older adults: study protocol for a three-arm randomized controlled trial |
| Countries of recruitment | Denmark |
| Health condition(s) or problem(s) studied | Physical frailty and pre-frailty conditions |
| Intervention(s) | PROT-group: protein supplementation to achieve 1.5 g/kg/day  EXEPROT-group: Power training 2 hours/week plus protein supplementation (1.5 g/kg/day)  REC-group: follow the European recommendations on protein intake and physical activity |
| Key inclusion and exclusion criteria | - Pre-frail or frail (SHARE-FI75+) - Community-dwelling - +80 years old - Medically stable (incl. kidney function, no allergies) - Intact cognitive function - Able to participate in group-based exercise - Signed informed consent |
| Study type | Randomized controlled trial with three arms |
| Date of first enrolment | 15.02.19 |
| Target sample size | 150 |
| Recruitment status | Recruiting |
| Primary outcome(s) | Lower leg muscle power |
| Key secondary outcomes | Muscle mechanical function, maximal muscle strength, physical function, mobility, physical Frailty, health-related quality of life, risk of malnutrition, activities of daily living, pain. |
